# Does early skin-to-skin contact have a long-term effect on the emotional and behavioral development of very preterm infants?

**DOI:** 10.3389/fpsyg.2024.1484419

**Published:** 2024-11-11

**Authors:** Patricia Trautmann-Villalba, Eva Heine, Angela Kribs, Katrin Mehler

**Affiliations:** ^1^Institute of Peripartal Interventions, Frankfurt, Germany; ^2^Department of Paediatrics, Faculty of Medicine and University Hospital Cologne, Cologne, Germany

**Keywords:** premature infant, preterm infant, skin-to-skin contact, behavior problems, school-age, parental stress, bonding, depressive symptoms

## Abstract

**Introduction:**

Premature birth may impair a sensitive, responsive, enjoyable, and regulating parenting style, potentially leading to behavioral, cognitive, and emotional deficits in children. Additionally, the emotional bond between the parent and infant may be disturbed due to the restrictions and difficulties at the neonatal intensive care unit (NICU), further negatively impacting child development. Skin-to-skin contact (SSC) directly after birth is strongly recommended also for preterm or low birth weight infants since there is high-certainty evidence that SSC has positive effects on neonatal and maternal health as well as on the quality of the parent–child relationship. The aim of this study was to examine the effect of skin-to-skin contact immediately after childbirth on the development of emotional and behavioral problems in children born preterm entering school.

**Methods:**

This study is part of a randomized controlled delivery room skin-to-skin study (*Deisy Study*). A total of 33 children (aged 6–8 years) were assessed at school start. The German version of the CBCL/6-18R was used to evaluate the presence of behavior problems.

**Results:**

The perceived parental stress 6 months after discharge was the variable that most contributed to the variance explanation. SSC immediately after childbirth was not significant in the prediction of emotional and behavioral problems at school start.

**Limitations:**

The study was conducted in a small study group. Partners' variables were not included. Information regarding sociodemographic variables and bonding quality was collected 6 months (corrected age) after birth. The measurement of children's behavioral problems is not objective and corresponds to the parents' perception.

**Clinical Trial Registration:**

https://clinicaltrials.gov, deisy study NCT01959737, deisy follow up NCT03366285.

## 1 Introduction

The idea that the birth of a child is one of the most fulfilling and satisfying experiences in life is widespread. However, reality shows that this assumption is not always true for all parents. The time during pregnancy and after birth is a very intense, stressful phase for all parents, and it is associated with a number of challenges, regardless of whether or not particular difficulties arise.

In the case of a preterm birth, that is, the birth that occurs before the 37th week of gestation (World Health Organization, 2023a,b), parents face a very critical period due to a double burden. They must manage the expected crisis of welcoming a new child while also dealing with the trauma of potential danger and separation due to the high-risk state of the newborn and potential admission to a neonatal intensive care unit (NICU) (Arizu et al., 2024). Prematurity is a well-known risk factor for long-term medical and developmental deficits such as sensory, motor, cognitive, and behavioral impairments and delays (Shaw et al., 2023; Twilhaar et al., 2018) but could also have a negative impact on parental mental health (Ionio et al., 2024; Roque et al., 2017) and on the parent–child relationship, including bonding, interaction, and attachment (Mira et al., 2024; Shaw et al., 2023).

These circumstances have an important effect on parents' emotional state that can lead to feelings of sadness, grief and loss, insecurity, a high level of stress, and depressive or anxiety symptoms regardless of the baby's clinical severity. Pisoni et al. (2019) found that mothers at the NICU reported elevated postnatal depressive symptoms and high stress levels, even when their preterm infants had low perinatal risk and normal neurological examinations. A recent systematic review of parental mental health studies following preterm birth (Sandnes et al., 2024) found that ~20% of the mothers of extreme or very low birth weight infants showed depression, anxiety, post-traumatic symptoms, and a high level of parenting stress in the first 2 years after birth. In this context, depressive symptoms appear to be the most common symptoms with rates as high as 40% in the early postpartum period among women with premature infants (Vigod et al., 2010). Parents of preterm babies are likely to experience up to 18 times higher mood disorders compared to parents of full-term babies (Carson et al., 2015; Helle et al., 2015). Even though most studies included only mothers, partners also experienced symptoms of depression and anxiety during the 12 first months and decreasing symptoms of post-traumatic stress disorder (PTSD) between 12- and 24-months corrected age (CA). However, the occurrence of these symptoms is lower in partners (Sandnes et al., 2024).

The very special emotional state that parents experience in the NICU, associated with the routines and conditions there, makes it difficult to establish and develop a parent–baby relationship. In a recent study, Ionio et al. (2024) found that feelings of tension, anger, and confusion experienced by the mother tend to negatively affect the quality of the bond with her child. With respect to interactive maternal behaviors, studies have shown that the experience in the NICU has a negative effect on these and the quality of the bond. This effect was mediated by the presence of depressive symptoms, the level of perceived stress (Gerstein et al., 2019), and not having had SSC immediately after childbirth (Mira et al., 2024). Even though there is no universally recognized description of the procedure, continuous and prolonged SSC initiated immediately after birth has been strongly recommended by the World Health Organization as routine care for all preterm and low birth weight infants after delivery since there is high-certainty evidence of its benefits (World Health Organization, 2022, 2023b). A number of positive effects on the baby's health, for example, the reduction of risk of neonatal death and the prevention of infections (Conde-Agudelo and Díaz-Rossello, 2016), the improvement of infants' vital signs (Zengin et al., 2023), or the reduction of pain (Campbell-Yeo et al., 2019), as well as on maternal health (Scime et al., 2019; Pathak et al., 2023) and on the quality of the parent–child relationship, have been described in the literature (Lilliesköld et al., 2023). Recently, an expert panel who developed a “Skin-to-Skin Pragmatic Implementation Guide” also highlighted the positive influence of SSC on an infant's sucking behavior, maternal breastfeeding self-efficacy and self-confidence, as well as the positive maternal perception of the breastfeeding birth parent (Brimdyr et al., 2023). In previous studies conducted in a German level III NICU, we have reported that a group of premature children who maintained SSC for 1 h immediately after birth showed a higher quality of mother–child interaction at 6 months (CA) compared to a group of children with similar characteristics who only maintained visual contact (VC) with their mothers for 5 min after birth. In addition, SSC mothers scored lower in depression scores and presented a better quality of bonding than those who only had VC (Mehler et al., 2020, 2024). Comparing the SSC group to a group of full-term infants and their mothers, SSC dyads showed similar interactional behaviors as term dyads. In contrast, the interactional behaviors in the VC group were significantly reduced (Heine et al., 2023).

Children born preterm are up to three times more likely to receive a psychiatric diagnosis at school age than those born full term. The most common diagnoses include emotional disorders, conduct disorders, attention-deficit/hyperactivity disorder, and autism spectrum disorder (Bhutta et al., 2002; Larsen et al., 2024; Treyvaud et al., 2014). In this context, temperament can play an important role: the meta-analysis conducted by Cassiano et al. (2020) supports the affirmation that preterm children present less regulated temperament than full-term children, which, in turn, could lead to a higher activity level and lower attentional focusing and persistence. In the same direction, Martins et al. (2021) reported that 18- to 36-month-old toddlers, who were born preterm and showed more negative affectivity, presented more behavior problems on the CBCL scores (total, externalizing, and internalizing) compared to full-term toddlers. This aspect is relevant since infant temperament is an independent risk factor for higher levels of parental stress (Gray et al., 2013). Parental stress, depression, and anxiety were found to be a significant and independent predictor of child cognitive and behavioral development of preterm infants, although most studies have focused on short-term rather than long-term effects (Greene et al., 2018; Mughal et al., 2017; Neri et al., 2017; Zelkowitz et al., 2011).

Due to the demonstrated benefits of SSC on the development of preterm infants and maternal mental health, we hypothesized that children who were born between the 25th and 32nd week of gestation and receive SSC in the delivery room (DR-SSC) may present lower levels of behavioral and emotional problems at the start of school, which may be irrespective of maternal depressive symptoms, bonding quality, and parental stress.

## 2 Materials and methods

### 2.1 Participants

**Figure 1:**
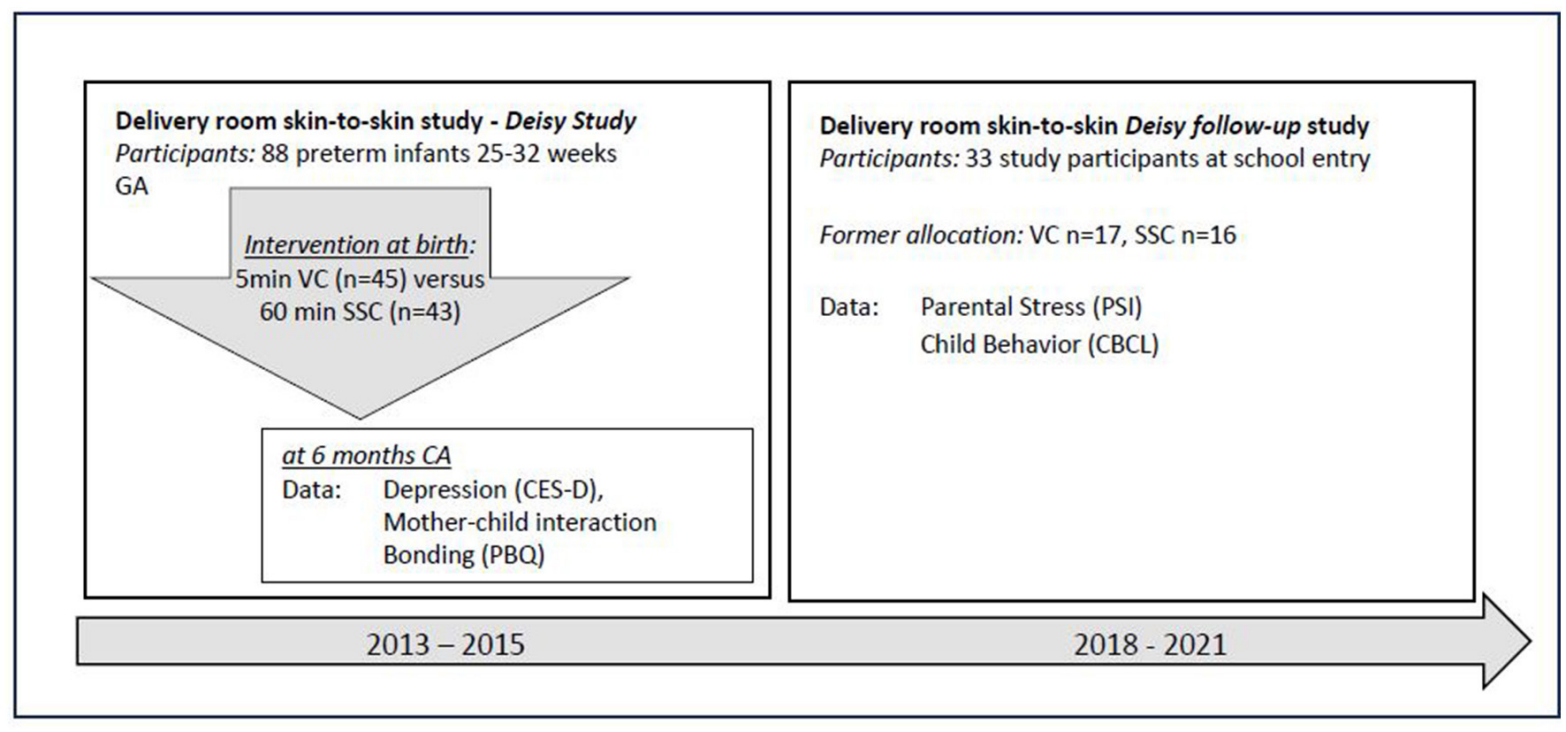
Timeline of the initial and the follow-up delivery room skin-to-skin study. GA, gestational age; VC, visual contact; SSC, skin-to-skin contact; CA, corrected age.

This study is part of a randomized controlled delivery room skin-to-skin study (*Deisy Study*, Mehler et al., 2020) conducted at a level III NICU at the University Children's Hospital of Cologne with the purpose of evaluating a standardized SSC intervention to improve the quality of the mother–child interaction and bonding. To enhance the neurodevelopment of these preterm infants and to reduce possible behavior problems, firstborn singletons between 25 and 32 weeks of gestation were included in the initial sample. After initial stabilization (approximately 45 min), the infant was transported to the room where the mother was cared for. In 2013, when the trial was conducted, neither physical nor short visual contact between parents and their preterm newborns was the standard of care in most neonatal departments. In our study, infants who received standard care had 5 min of visual contact with their parents. Visual contact allowed them to see but not to touch or kiss their infant. Dyads randomized into the intervention group received 60 min uninterrupted SSC, supervised by the neonatal team (detailed information in Mehler et al., 2020). Eighty-eight newborns constituted the initial sample (DR-SSC *n* = 45, VC *n* = 43). Two data collections were planned: at 6 months corrected age (CA) and between 6 and 8 years, the time when children enter school, to assess the long-term effects of the DR-SSC on their behavior. Data collection took place between 2018 and 2021. Figure 1 shows the design of the initial and the follow-up trial, and the respective data collection used in this study.

From 2020, the restrictions due to the COVID-19 pandemic made home visits difficult. Therefore, the study group was greatly reduced. No significant differences were found between participants at the 6- to 8-year follow-up and non-participants regarding neonatal characteristics, maternal age and educational variables, family income, single parenthood, maternal depressive symptoms at child's discharge, nor in the quality of bonding or parental stress at the age of 6 months. In the study at school start, 33 children participated (SSC *n* = 16, VC *n* = 17). The sociodemographic and neonatal characteristics of the sample are shown in Table 1.

**Table 1:** Characteristics of the study group and their mothers (*N* = 33)^a^.

	**VC**	**SSC**	***p***
Gender Female (%)	10 (58.8)	10 (62.5)	0.556
Gestation (weeks)	29.2	28.8	0.550
Birth weight (in grams)	1,143.6	1,236.9	0.513
10-min Apgar score	8.47	8.50	0.884
Days at the hospital	60.1	63.8	0.691
Firstborn (%)	11 (68.8)	10 (62.5)	0.592
Age-appropriate school enrollment (%)	12 (70.6)	12 (75.0)	0.859
Maternal age	37.8	39.2	0.480
High school degree (mother)	13 (81.3)	15 (93.8)	0.300
Professional qualification (mother)	15 (93.8)	16 (100)	0.500
Single parent	1 (5.9)	3 (18.8)	0.335
Perceived social support	4.5	4.4	0.824
CES-D	10.1	12.6	0.480
PSI total score	55.1	55.8	0.871
PBQ total score	10.7	9.1	0.582

^a^N and % for nominal variables/M for continuous variables.

CES-D, Center for Epidemiological Studies Depression Scale; PSI, Parental Stress Index; PBQ, Postpartum Bonding Questionnaire.

The study was approved by the Ethics Committee of the University of Cologne and was conducted in accordance with the 1964 Declaration of Helsinki. The study is registered under the number NCT03926923.

### 2.2 Instruments

To identify depressive symptoms, quality of bonding, parental stress, and child behaviors, self-reporting methods were used. The assessment time is presented in Figure 1. Sociodemographic data (e.g., parental age, level of education, financial situation, employment, and number of children) and basic data on neonatal course were collected at birth.

#### 2.2.1 Center for epidemiological studies depression scale (CES-D)

Depressive symptoms were assessed with the German version of CES-D, long form (Hautzinger and Bailer, 1993). This instrument consists of 20 self-descriptive statements in the first person relating to the past week. The items refer to typical affective, cognitive, somatic, and social depressive symptoms. The possible answers range from “0—rarely/not at all” to “3—mostly/all the time”. All 20 items added together build a total score (range of 0–60), and 4 items are scored reversely. Higher values indicate a higher level of depressive symptoms. Clinical cutoffs are also available. For this study, only the total value is used. The German version of CES-D has good overall psychometric proprieties and internal reliability (Cronbach's alpha 0.85–0.92).

#### 2.2.2 Postpartum bonding questionnaire (PBQ)

The PBQ was originally developed to assess the emotional bond between a mother and her child (Brockington et al., 2001, 2006), but meanwhile, the PBQ has been also frequently used for partners. The PBQ is a self-report screening instrument and consists of 25 positive and negative statements rated on a scale of 0 to 5. Positive statements are scored as “marked” and negative statements as “reversed”. The sum of all values constitutes a general score; in addition, three subscales could be built: impaired bonding, rejection and anger, and anxiety about care. For all scales, a higher score indicates more bonding difficulties. The German version yields a good internal consistency (Cronbach's alpha 0.85) (Reck et al., 2006). Brockington et al. (2001) proposes cutoff points for each scale to identify problematic bonding for this study; however, we only used the scores.

#### 2.2.3 The parenting stress index (PSI)

The PSI (Abidin, 1995) is a widely used instrument to assess stress specifically associated with parenting. The self-report items are related to the parent's perception of difficulties in the parental role, the parent–child interaction, and specific infant characteristics, which make it difficult to cope with the child (e.g., the baby is perceived as irritable, agitated, and moody). The German version (Hofecker Fallahpour et al., 2009) contains 12 subscales with four items each. Items are scored on a 5-point Likert scale. The scales indicate the source of stress and cover the following aspects: distractibility/hyperactivity, mood, acceptability, demand, and adaptability in the child domain and attachment, social isolation, parental competence, depression, health, personal limitations, and partner relationship in the parent domain. Two partial scores and total scores could be built. Although cutoff values were established to identify parents with significant levels of distress, only the total score values were included in the analysis. The German version demonstrated very good internal consistency, with a Cronbach's alpha of 0.95 for the total score and 0.91 to 0.93 for the subscales.

#### 2.2.4 Child behavior checklist/6-18 (CBCL/6-18)

The Child Behavior Checklist/6-18 is an internationally widely used, standardized tool for assessing behavioral and emotional problems of school-age children (6–18 years). The German version (Döpfner et al., 2024) consists of a 118-item questionnaire and is administered to parents using a 3-point scale (0 = not true to 2 = very true) to rate how true each item is. Scores could be calculated for eight clinical subscales (Withdrawn/Depressed, Somatic Complaints, Anxious/Depressed, Rule-Breaking Behavior, Aggressive Behavior, Social Problems, Thought Problems, and Attention Problems). These subscales can be grouped into two higher-order factors: internalizing and externalizing problems (Cronbach's alpha of 0.90 and 0.94, respectively) and added up to a total problem score (Cronbach's alpha of 0.97).

### 2.3 Statistical analysis

For data management and statistical analysis, SPSS (Version 28, IBM, Armonk, NY, USA) was used. Pearson's chi-square and univariate ANOVA were used to test the similarity of the study group with the drop-out group, likewise to prove the homogeneity among the SSC and the VC groups.

Means and standard deviations for the CES-D, PBQ, and PSI scores were calculated, and Spearman's rho coefficients were used to determine the association between these variables (CES-D, PSI, and PBQ) as well as the association between the variables and the CBCL scores.

A stepwise hierarchical regression analysis was performed to assess the significance of DR-SSC in the prediction of emotional and behavioral problems (CBCL total score) while controlling for neonatal course and maternal variables, including depressive symptoms, parental stress, and bonding. In the first step, maternal age and child gender were included, followed by the APGAR score at 10 min after birth and the number of hospitalization days. After that, the CES-D score, the PBQ, the PSI variables, and finally skin-to-skin contact were introduced (forced) into the model.

The level of significance for all tests was two-sided and set at a *p* = 0.05.

## 3 Results

### 3.1 Depressive symptoms, parental stress, and bonding

Descriptive statistics for depressive symptoms, parental stress, and bonding (at 6 months CA) are presented in Table 1. Maternal depressive symptoms were significantly correlated only with the parent domain score of the PSI. On the other side, higher scores in the child domain were associated with more rejection and anger and more anxiety about care. Interestingly, the only PBQ scale that correlated with the PSI parent domain was impaired bonding. Both domains were strongly associated. Contrary to our expectations, no PBQ scale correlated significantly with depressive symptoms. Furthermore, higher parental stress (PSI total score) is associated with a poorer maternal bond to her child (PBQ total scores). All results are shown in Table 2.

**Table 2:** Descriptive statistics and intercorrelations (6 mo. CA).

	**M**	**SD**	**Range**	**1**.	**2**.	**3**.	**4**.	**5**.	**6**.	**7**.
1. CES-D	10.5	8.95	0–35	-					
2. PSI Child Domain	57.7	10.22	32–70	0.219	-				
3. PSI Parent Domain	54.52	9.61	30–70	**0.375** ^*^	**0.883** ^***^	-			
4. PSI Total Score	56.06	10.58	30–70	0.310	**0.957** ^***^	**0.970** ^***^	-		
5. PBQ Impaired bonding	5.57	4.37	0–15	0.264	0.342	**0.362** ^*^	0.340	-	
6. PBQ Rejection and anger	2.23	3.06	0–14	0.247	**0.442** ^*^	0.242	0.340	**0.728** ^***^	-
7. PBQ Anxiety about care	2.07	1.62	0–7	0.068	**0.442** ^*^	0.341	**0.380** ^*^	**0.448** ^*^	**0.464** ^*^	-
8. PBQ Total Score	9.87	7.7	0–34	0.219	**0.475** ^**^	**0.393** ^*^	**0.424** ^**^	**0.922** ^***^	**0.872** ^***^	**0.631** ^***^

^***^p <0.001; ^**^p <0.01; ^*^p <0.05.

CES-D, Center for Epidemiological Studies Depression Scale; PSI, Parental Stress Index; PBQ, Postpartum Bonding Questionnaire. Bold means statistically significant.

### 3.2 CBCL scores and maternal depression, parental stress, and bonding at school entry

No significant associations were found between maternal depressive symptoms and any CBCL score. However, both PSI domains (parent and child) presented a high level of association with all CBCL scores: Higher perceived stress was associated with a higher number of externalizing and internalizing behaviors, with a stronger association observed for externalizing behaviors. In relation to the values observed with the PBQ, no scale presented a significant association with internalizing behaviors, while the impaired bonding scale and the PBQ total score correlated with the externalizing and the total score. The CBCL total score was strongly associated with all aspects of the PSI and the PBQ (Table 3).

**Table 3:** Descriptives for CBCL scores and correlation with CES-D (Center for Epidemiological Studies Depression Scale), PSI (Parental Stress Index), and PBQ (Postpartum Bonding Questionnaire).

**Variables**	**CBCL internalizing**	**CBCL externalizing**	**CBCL total score**
1. CES-D	0.147	0.164	0.116
2. PSI child domain	0.362^*^	0.623^***^	0.802^***^
3. PSI parent domain	0.348^*^	0.598^***^	0.687^***^
4. PSI total score	0.394^*^	0.607^***^	0.748^***^
5. PBQ impaired bonding	0.241	0.455^*^	0.433^*^
6. PBQ rejection and anger	0.163	0.295	0.390^*^
7. PBQ anxiety about care	0.136	0.317	0.380^*^
8. PBQ total score	0.290	0.446^*^	0.511^**^
M (S)	56.61 (6.55)	54.33 (9.91)	57.36 (8.55)
Min–Max	45–70	35–73	40–74
>cutoff	*N* = 5	*N* = 3	*N* = 5

N = 33.

^***^p <0.001; ^**^p <0.01; ^*^p <0.05.

### 3.3 The role of skin-to-skin contact immediately after childbirth in the prediction of emotional and behavioral problems at school start

Maternal age, gender of infant, Apgar score, hospitalization days, and maternal depressive symptoms—included in the analysis for control—were not significant in any of the models. Even though the PBQ total score reached significance in the first model, this significance was lost in the next step, when the PSI variables were entered. The PSI child domain was the only variable that attained significance and remained significant after the forced inclusion of DR-SSC, which was not significant. The last model explained 66% (R^2^ 0.661, R^2^cor 0.621) of the variance (<0.001). The results of the stepwise regression analysis are shown in Table 4.

**Table 4:** Skin-to-skin contact and Child Behavior Checklist Score at school start—stepwise (forward) regression analysis.

**CBCL total score**						
	**ß**	**T**	**R** ^2^	**R**^2^**adj**.	**F**	**p**
**Model 1**						
PBQ total score	0.418	2.394				
			**0.175**	(0.145)	5.731	**0.024**
**Model 2**						
PBQ total score	0.084	0.667				
PSI child domain	0.773	6.107				
			**0.661**	(0.635)	25.365	**<0.001**
**Model 3**						
PBQ total score	0.087	0.657				
PSI child domain	0.772	5.931				
DR-SSC	−0.010	−0.083				
			**0.661**	(0.621)	16.267	**<0.001**

Excluded variables: maternal age, gender of infant, 10-min Apgar score, hospitalization days, and maternal depressive symptoms.

PBQ, Postpartum Bonding Questionnaire; PSI Parental Stress Index; DR-SSC, delivery room skin-to-skin contact. Bold means statistically significant.

## 4 Discussion

The aim of this study was to examine the effect of SSC immediately after childbirth on the development of emotional and behavioral problems in children born preterm entering school. It was hypothesized that children receiving DR-SSC would have fewer behavioral and emotional problems than children receiving only VC in the delivery room. We expected that the positive effect of SSC observed by different authors and in our own previous studies could also be maintained in the long term. However, this hypothesis could not be demonstrated: SSC immediately after childbirth was not significant in the prediction of emotional and behavioral problems at school start. Neither the depressive symptoms nor the quality of bonding variable contributed the most to the variance explanation, but the perceived parental stress at 6 months CA variable did, which was an unexpected result. A fundamental aspect of the interpretation of the results is the small sample size, which unfortunately does not allow for more complex analyses and can mislead the results.

The fact that DR-SSC was not the explanatory factor for the behavioral and emotional problems observed in school-aged children could be understood (among others), through the feeling of being a family and belonging together that first becomes evident to parents as time passes—a comment that is often heard in contact with these parents in daily clinical practice. Parents of premature children often say that they do not perceive this feeling of belonging until the moment their children are discharged, unlike parents of children born full term and without problems, who can engage in the parental role and, for example, hold their children immediately after birth (Schuetz Haemmerli et al., 2020). Therefore, DR-SSC, an intervention that has a high impact at that specific moment in family life, might lose this effect over time and with the adjustment of parental feelings and family life.

The lack of association between maternal depressive symptoms and the level of behavioral and emotional problems of children at school entry could be explained in the same direction. Although depressive symptoms have been widely demonstrated to be a risk factor for the presence of children's behavioral and emotional problems (even regardless of prematurity), it is important to consider that the depressive data we used for our analyses were collected a long time before. The existing literature for the specific case of parents of premature children indicates that in most cases, depression gradually declines over time (Barkmann et al., 2018; Genova et al., 2022; Pace et al., 2016). This trend was also observed in our study (Mehler et al., 2020). However, depressive symptoms remain associated with parental stress (parent domain), a phenomenon that could be explained by the inverse relationship between parents' perception of the demands of parenting and the personal available resources to cope with them. This lack of confidence has been described even for depressive parents of full-term children (Aviles et al., 2024; Pazzagli et al., 2023; Reck et al., 2016; Sandnes et al., 2024).

Similarly, parental stress plays an important role in the development of an emotional bond with the baby: The higher the stress perceived by the birth parents, the worse the bond quality to their preterm infants. Our results showed a strong association between the Parental Stress Index with the observed values of the PBQ at 6 months. The child domain score, which comprises negative characteristics of the infants, is related to the PBQ-Subscales Rejection and Anger, Anxiety about care, and the PBQ Total Score. It could be hypothesized that these children were already more irritable, agitated, moody, etc. at the age of 6 months and that is why their mothers had difficulties in establishing a good bond with them, which in turn would allow us to think that these negative characteristics continue even at the age of entering school. In addition, the PBQ-Subscale Impaired Bonding, which indicates some kind of occasional delay in, or temporary loss of maternal feeling about the infant but not established rejection or anger (Brockington et al., 2001, 2006), is the only subscale associated with the parent domain score. Further maternal bonding and parenting stress were still significantly associated with CBCL scores at school beginning but not with depressive symptoms. These results are in line with those by Fransson et al. (2020), who pointed out the mediating role of postpartum bonding difficulties in this context. This association between parental stress and PBQ scores could explain the loss of significance when the parental stress variable was entered into the regression model, although the bonding quality plays an important role in the development of behavioral problems.

Finally, the role of perceived parental stress could be better understood from a differentiated perspective of parental stress in the context of prematurity and the COVID-19 pandemic. Chronic but also acute stressors have the potential to increase the demands of parenting and thus parenting stress (Aviles et al., 2024). In their review, Sandnes et al. (2024) reported increased stress levels in the first 2 years after birth in 15–56% of parents of premature infants; however, only a few studies have focused on parental stress into school age or longer. Nevertheless, parenting stress following preterm birth can remain high until the age of 7 years and could be mainly caused by children's difficulties (Treyvaud et al., 2014) or characteristics, such as temperament or chronic physical and/or mental health conditions (Deater-Deckard and Panneton, 2017). In any case, parenting stress can increase the risk of a child's negative behavioral and emotional outcomes in the present and/or over the years, certainly not only by children born preterm (Crnic et al., 2005; Deater-Deckard and Panneton, 2017; Han and Lee, 2018; Neece et al., 2012; Ribas et al., 2024; Spinelli et al., 2020). Considering that part of the data related to children's problem behaviors was carried out during the COVID-19 pandemic, it is possible to speculate that this high-stress situation had an impact on both the level of parental stress and the CBCL scores. All of this allows us to hypothesize that the level of parental stress in these families could have remained high in these years and thus explain the strong effect of this variable on the behavioral problems of children upon entering school. In addition, this fact may have led to a selection bias in the group that participated in this part of the study. On the one side, parents could have facilitated participation in the study to obtain a founded professional opinion if they have noticed an increase in problematic behaviors by their children or, on the other, to address doubts and obtain specialized guidance if necessary.

## 5 Limitations

Clear limitations of this study should be mentioned. Even though the study group does not differ in relation to sociodemographic and perinatal variables from the original group, the participating families represent <40% of it. A major deficit was not having included information about the partner (as a possible moderating factor) and not having collected current information. Furthermore, the children's behaviors were only evaluated with a global instrument (CBCL/6-18), which does not collect objective information but instead the perception of the parents. One can speculate that the DR-SSC could have long-term effects on other areas of development or that its effect could be much more differentiated than that which could be assessed with the instruments used.

## 6 Conclusion

The extensive existing literature has shown that DR-SSC, even for very premature children, is safe and feasible and has important benefits for both the babies and their parents. Although in this study its long-term effect could not be demonstrated in relation to the presence of behavioral problems upon entering school, other results are still interesting. Parental stress and its long-term influence on the emergence of behavioral problems is an important piece of information, especially for the support and follow-up programs for families with children born prematurely. In the long-term perspective, maternal stress seems to be much more important than having had skin-to-skin contact immediately after birth. Since premature birth is a situation of extreme vulnerability for the entire family, a comprehensive and careful approach that also includes psychoeducation, coping with stress, parental empowerment, and at least family mental health is required. Since in Germany, there are numerous care programs for premature children and their families, the affected families normally receive the necessary support throughout the different evolutionary stages. Medical, psychological, financial, and social familial support is guaranteed by law.

## Data Availability

The raw data supporting the conclusions of this article will be made available by the authors, without undue reservation.
